# Beyond the classical Fc receptor: CD64 in phagocytosis and trained immunity

**DOI:** 10.3389/fimmu.2026.1985821

**Published:** 2026-10-20

**Authors:** Zhaoxia Qu, Hongqiao Zhang, Steven D. Shapiro, Gutian Xiao

**Affiliations:** 1 Department of Immunology and Immune Therapeutics, Keck School of Medicine, University of Southern California, Los Angeles, CA, United States; 2 Hastings Center for Pulmonary Research, Keck School of Medicine, University of Southern California, Los Angeles, CA, United States; 3 USC Norris Comprehensive Cancer Center, Keck School of Medicine, University of Southern California, Los Angeles, CA, United States; 4 Division of Pulmonary, Critical Care and Sleep Medicine, Department of Medicine, Keck School of Medicine, University of Southern California, Los Angeles, CA, United States

**Keywords:** CD64/FcγRI, C-reactive protein (CRP), epigenetics, innate immune memory, metabolism, NF-κB, pattern recognition receptor (PRR), pentraxin (PTX)

## Abstract

CD64 (FcγRI) is traditionally categorized as the high-affinity receptor for immunoglobulin G (IgG) and as a downstream effector of adaptive humoral responses for myeloid-driven clearance and immune programs. Accumulating evidence in myeloid cell biology, however, highlights a major historical oversight: CD64 also operates as a multimodal innate immunoreceptor capable of antibody-independent pattern recognition receptor (PRR) behavior. Synthesizing over three decades of data, this review spotlights the direct interaction of CD64 with humoral pentraxins and microbial outer membrane structures independent of IgG opsonization. We consolidate the biological mechanisms by which CD64 functions as an opsonin receptor or a direct PRR to drive phagocytosis and immune signaling pathways. Because trained immunity is antibody-independent, we introduce an unaddressed paradigm for how CD64 utilizes its non-classical, primordial PRR identity to guide myeloid cells to establish and maintain innate immune memory. This framework resolves a profound evolutionary and teleological conundrum: why frontline myeloid lineages constitutively express and exponentially upregulate CD64 days before pathogen-specific antibodies are manufactured, leveraging an ancient, non-specific pattern-sensing scaffold. Finally, we conceptualize the multi-tiered regulation of CD64 as a precise, localized translational target and predictive biomarker to safely bypass the potential risks of global metabolic and epigenetic therapies.

## Highlights

- A dual-identity immunoreceptor: CD64 transcends its textbook classification as a passive IgG receptor, functioning autonomously as an antibody-independent PRR to correct a major historical blind spot.
- Direct ligand engagement: CD64 directly binds microbial outer membrane structures and humoral pentraxins independent of canonical, antibody-mediated IgG opsonization.
- Intracellular signal execution: Beyond simple binding, this non-classical innate receptor actively coordinates multi-tiered regulatory networks to drive autonomous phagocytosis and downstream inflammatory signaling pathways.
- Orchestrating trained immunity: Positioned as a central driver of antibody-independent innate memory, CD64 guides myeloid cells through induction, immunometabolic imprinting, epigenetic reprogramming, and hyper-responsive recall of trained immunity.
- Evolutionary host defense: Frontline myeloid lineages inherently express and meteorically upregulate CD64 days before B cells produce antibodies, resolving evolutionary and teleological puzzles by leveraging an ancient, non-specific pattern-sensing scaffold.
- Translational outlook: Conceptualizing CD64 as a precise, localized therapeutic target and predictive biomarker offers a theoretical framework to bypass the potential risks of global metabolic and epigenetic therapies.

## 1 Introduction

The classical view of immunity rigidly separates innate and adaptive responses (1). In this binary framework, innate immune cells—particularly macrophages—recognize invading pathogens via canonical pattern recognition receptors (PRRs) to execute phagocytosis, kill the pathogen, and initiate inflammation while presenting processed antigens to prime adaptive immunity. This cascade ultimately culminates in the activation of B cells and the production of highly specific antibodies spanning diverse immunoglobulin (Ig) classes. Historically, Fc receptors (FcRs) were discovered through their direct binding affinity for the fragment crystallizable (Fc) region of IgG molecules in 1980s; yet since then they have been conceptualized merely as downstream accessories to adaptive immunity for more potent, high-velocity, and antigen-specific pathogen clearance (2–8). Among these diverse receptor systems, CD64 (FcγRI) occupies a unique position as the only high-affinity receptor capable of binding monomeric IgG, the predominant and most versatile antibody class driving systemic humoral immunity (8, 9). Consistently, CD64 is predominantly expressed by myeloid cells, particularly phagocytic macrophages, which serve as the primary cellular sentinels defending against pathogens (10, 11).

However, this strictly downstream, antibody-dependent model introduces a profound teleological puzzle regarding cell-surface expression dynamics. If classical FcRs and CD64 function exclusively after a matured adaptive response has developed, why do frontline sentinel macrophages and most myeloid lineages constitutively and inherently express these receptors prior to any initial pathogen contact? Furthermore, upon acute infection, their expression is meteorically upregulated—such as the massive, rapid mobilization of CD64 on neutrophils, which maintain low baseline levels in a resting state (12–19). This high-energy, early-phase surface deployment occurs days before B cells can manufacture a single pathogen-specific Ig molecule. These facts strongly indicate that this receptor possesses an unappreciated, intrinsic purpose during the initial hours of a primary infection.

Answering this enigma is an accelerating body of evidence demonstrating that myeloid cells can mount enhanced responses to secondary insults entirely independent of adaptive lymphocytes (20, 21). Concurrently, our understanding of CD64 biology has expanded past its classical boundaries (22). Crucially, CD64 may occupy a far superior position in immune regulation than canonical PRRs. Rather than acting as a simple passive sensor, CD64 functions as a multi-modal hub: besides being primed by classical IgG, it handles innate opsonins like fluid-phase pentraxins, complexes cooperatively with conventional PRRs like Toll-like receptors (TLRs), and may even act as a direct, dynamic PRR itself to recognize and bind to bacterial outer-membrane structures independent of immunoglobulins. In this review, we propose a novel conceptual framework exploring the dual nature of CD64, hypothesizing its unaddressed role as a potential master driver, maintainer, amplifier, and/or executor of trained immunity.

## 2 Divergent activation of CD64

While CD64’s identity as a high-affinity IgG receptor has been universally recognized since the 1980s (1–4, 6–8), its parallel capacity to operate as an autonomous or cooperative PRR remains systematically marginalized, frequently dismissed by automated literature syntheses and AI knowledge graphs that flatten it only as the IgG receptor FcγRI. Far from a nascent discovery, this innate axis was established thirty-five years ago when a human monocytic cell line was shown to utilize CD64 to capture C-reactive protein (CRP) in 1991—a foundational milestone subsequently validated and extended by independent cohorts demonstrating that CD64-mediated binding of CRP, serum amyloid P component (SAP), or pentraxin 3 (PTX3) actively drives phagocytosis (23–40). The innate opsonin pathway was structurally cemented by crystallographic mapping, proving that humoral pentraxins directly engage and cross-link the classical Fc-binding domains of the receptor in a completely antibody-independent manner (31). Modern biophysical insights further reveal that the interaction is strictly regulated by the conformational state of the ligand (40). Intriguingly, the receptor’s innate programming expands beyond soluble opsonin trapping into direct pathogen sensing. *Escherichia coli* K1 completely bypasses immunoglobulin shielding by directly hijacking CD64 via its outer membrane protein A (OmpA) to survive within macrophages, build high-grade bacteremia, and breach the blood-brain barrier (41). However, because this host-pathogen interaction was reported solely by a single research group over sixteen years ago and has yet to be replicated by independent laboratories, further validation is required to establish its physiological relevance. Independent replication is particularly important given that the reported mechanism of CD64 activation exhibits a distinct logical inconsistency with standard receptor biology, as detailed below. Nevertheless, from an evolutionary standpoint, such multi-modal, antibody-independent behavior is entirely logical. Fc receptors share an ancient phylogenetic heritage with primitive, non-specific pattern-sensing scaffolds in lower vertebrates that bound microbial carbohydrates and lipids long before the evolutionary emergence of adaptive immunoglobulin loops (42–44). Thus, when CD64 reacts to pentraxins or bacterial outer membranes without an antibody intermediary, it is deploying its deeply conserved, primitive innate immunity programming.

### 2.1 Structural topology of the CD64 extracellular domain

The extracellular protein architecture of CD64 (FcγRI) is unique from other Fcγ receptors and consists of three C2-type immunoglobulin-fold domains, designated as D1, D2, and D3 (8, 45, 46). X-ray crystallography and solution-state structures confirm that the N-terminal D1 and D2 domains form an acute, tight interface containing the primary hydrophobic binding cleft responsible for raw ligand engagement (47, 48). In contrast, the unique membrane-proximal D3 domain does not directly touch immunoglobulins (46). Instead, D3 operates as a vital conformational stabilizer and rigid spacer that locks the D1-D2 interface into an optimal spatial orientation, effectively blocking fast thermal dissociation (48). This structural configuration provides CD64 with its characteristic 100- to 1,000-fold higher binding affinity than its two-domain family members, CD32 and CD16 (48, 49).

### 2.2 Classical opsonin receptor for IgG engagement

Under steady-state conditions, CD64 coordinates adaptive humoral immunity by capturing the constant Fc region of IgG (8). The lower hinge region of the IgG *C_H_2* domain inserts directly into the interface cleft of the D1 and D2 domains (45–48). A conserved hydrophobic pocket located in the D2 domain provides a precise structural fit for specific amino acid residues (such as Leu235) protruding from the IgG Fc stem (8, 48). Because of the structural rigidification provided by the D3 domain, this molecular pocket is stable enough to trap individual, monomeric IgG molecules out of fluid circulation (*K_D_* ≈ 10–100 nM) even in the complete absence of antigen cross-linking (**Table 1**).

**Table 1:** Characteristics of CD64 binding to IgG and pentraxin.

Biochemical attribute	Classical	Non-Classical
Ligand	IgG	Pentraxins
Ligand Structural Geometry	Linear, flexible dimeric Fc stem	Rigid, planar symmetric pentameric disc
Binding Kinetic Affinity	High affinity (K_D_ ≈ 10^–9^ M); traps monomers	Moderate affinity (K_D_ ≈ 10^–6^ M); relies on avidity
Primary Domain	Interface cleft of D1 and D2	Interface cleft of D1 and D2
Receptor Clustering Logic	Driven by IgG complexes cross-linking	Induced instantly by the multivalent pentameric ligands
Competitive Inhibition	Blocked by anti-CD64 or excess pentraxins	Completely displaced by monomeric human IgG
Common signaling core	Phosphorylation of common γ-chain ITAM	Phosphorylation of common γ-chain ITAM

### 2.3 Non-classical opsonin receptor capturing innate fluid-phase pentraxins

Beyond its textbook adaptive identity, CD64 handles innate, antibody-independent opsonophagocytic defense by directly binding fluid-phase pentraxins (PTX) CRP, SAP, and PTX3—pattern recognition molecules made by the liver or locally by macrophages and endothelial cells that bind to microbes and damaged cells (22–40).

During the acute, antibody-naive phase of a primary infection, the liver rapidly secretes CRP, a prominent, planar, symmetric pentameric pattern recognition protein that interacts with many pathogen- or damage-associated patterns (22, 50). Biochemical and live-cell imaging studies show that pentameric CRP directly docks onto the D1 and D2 domain interface of CD64 with micromolar affinity (*K_D_* ≈ 6.6-10 μM) (31). This interaction matches the exact binding footprint of monomeric IgG, causing the two ligands to physically compete for the same receptor real estate. Although monomeric IgG maintains a thermodynamically superior baseline affinity, a massive acute-phase concentration surge of CRP (up to 1,000-fold, >100 mg/L) drives mass-action competition for the receptor cleft (22, 50). Once docked, the unique physical shape of the ligand alters cell-surface behavior; unlike the linear, flexible stem of IgG, the pentraxin molecule presents a wide, multivalent, rigid disc that physically bridges and cross-links multiple adjacent CD64 molecules simultaneously. This spontaneous receptor clustering shifts the binding dynamic from a weak monomeric interaction to a high-avidity platform, slowing down the ligand dissociation rate to near zero (22, 46–49). This biophysical clustering perfectly mimics the cross-linking effect typically triggered by an antigen-bound antibody network, generating a downstream activation signal that matches the functional potency of an adaptive response.

Other fluid-phase pentraxins, including SAP, utilize similar planar, hydrophobic disk geometries to bind the CD64 platform, enabling the receptor to flag pathogens and exposed host nuclear remnants for phagocytic disposal before B cells produce a single antibody (22).

### 2.4 Direct PRR for pathogen components

CD64 was proposed to function as a direct, dynamic PRR that did not rely on fluid-phase opsonin intermediaries to sense microbial invaders (41). According to this model, its extracellular framework directly recognized and physically bound to OmpA of Gram-negative pathogens, such as E. coli K1, independently of immunoglobulins or complement cascades (41). This reported opsonin-independent capture of a structural microbial cell wall component suggested that the structural framework of CD64 retained an evolutionary ancestral PRR identity (42–44).

Biophysically, however, the reported parameters of this direct OmpA engagement introduced a stark logical inconsistency when contrasted with standard receptor biology. A single *E. coli* cell typically displays a massive density of OmpA, presenting approximately 100,000 molecules on its outer membrane (51). In standard receptor models, encountering such a hyper-abundant, tightly packed lattice of surface ligands would inevitably trigger extensive, multi-valent receptor clustering and robust cross-linking. Yet, the direct OmpA-CD64 engagement failed to cross-link CD64 to activate the associated common γ-chain homodimer, instead bypassing this standard pathway to trigger an independent pattern of direct CD64 tyrosine phosphorylation cascades (41).

While independent validation remains required to substantiate this atypical and uncharacterized biophysical phenomenon, it is well established that certain pathogens have evolved to hijack host cellular signaling or even defense mechanisms—especially PRRs—to facilitate intracellular entry and exploit the host cell (52–54). It therefore remains highly plausible that CD64 naturally operates as a standalone, direct PRR tissue sentinel irrespective of its reported interaction with OmpA. This intrinsic capability would allow frontline macrophages to rapidly engulf and survey unopsonized invaders during the immediate phase of a primary encounter. Hence, characterizing whether—and through what structural mechanisms—CD64 recognizes other conserved pathogen-associated molecular patterns (PAMPs) represents a critical and entirely open frontier in innate immunobiology.

### 2.5 Non-classical opsonin receptor behavior of CD64 family members

The evolutionary landscape of antibody-independent sensing extends across the broader low-affinity FcγR network, where CD32 (FcγRII) and CD16 (FcγRIII) mirror CD64’s multi-modal PRR behavior by directly engaging innate pentraxins like CRP, SAP, and PTX3 (22, 25–29, 31, 32, 34, 36–40, 55). However, because CD32 and CD16 possess only two extracellular domains (D1 and D2) and lack the rigidifying D3 domain unique to CD64, they are biophysically constrained to utilize distinct structural binding mechanisms (22). Consequently, these two-domain platforms require specific polymorphic loop variations or localized, conformation-dependent pentraxins isoforms (such as pCRP*) to effectively stabilize and cross-link the receptor interface (22, 40). Nevertheless, this unified architecture ensures that prior to adaptive antibody maturation or when a pathogen actively evades established humoral recognition, the integrated FcγR network acts collectively as a frontline, multi-receptor PRR framework to drive phagocytosis and downstream immune signaling pathways.

## 3 Pathogen-induced intracellular signaling via CD64

Structurally, CD64 possesses a short cytoplasmic tail (46, 47). To bridge extracellular engagement to active cell machinery, the receptor usually non-covalently couples with a signaling homodimer composed of the immunoreceptor tyrosine-based activation motif (ITAM)-bearing common γ chain (22, 46, 47, 56). While classical and non-classical opsonin pathways feed into a unified proximal tyrosine kinase core, their downstream pathways may diverge in the strength and how they cross-talk with other innate immune sensors to execute highly separate, context-dependent biological functions (22, 57, 58). Moreover, this shared intracellular core is completely uncoupled during direct, antibody-independent PRR activation by unopsonized pathogens (41).

### 3.1 Common core signaling node as an opsonin receptor

Regardless of whether the activating ligand is multivalent, pathogen-bound IgG or the innate fluid-phase pentraxin CRP complexed with a pathogen, early structural engagement forces CD64 to recruit a shared proximal enzymatic machinery (22, 56). This core node is initiated by triggering Src-family tyrosine kinases (predominantly Lyn and Fyn) to phosphorylate the conserved dual tyrosine residues within the associated common γ-chain’s ITAMs. Most likely, with the help of other pathogen- and phagocytosis-related signaling pathways, this phosphorylated platform forms a docking station that binds the tandem SH2 domains of spleen tyrosine kinase (SYK), recruiting the p85 regulatory subunit of PI3K to activate multiple signaling pathways (59–62). Through this common γ-chain-dependent entry point, pathogen-loaded IgG and CRP drive overlapping foundational baseline functions (**Figure 1**):

**Figure 1:**
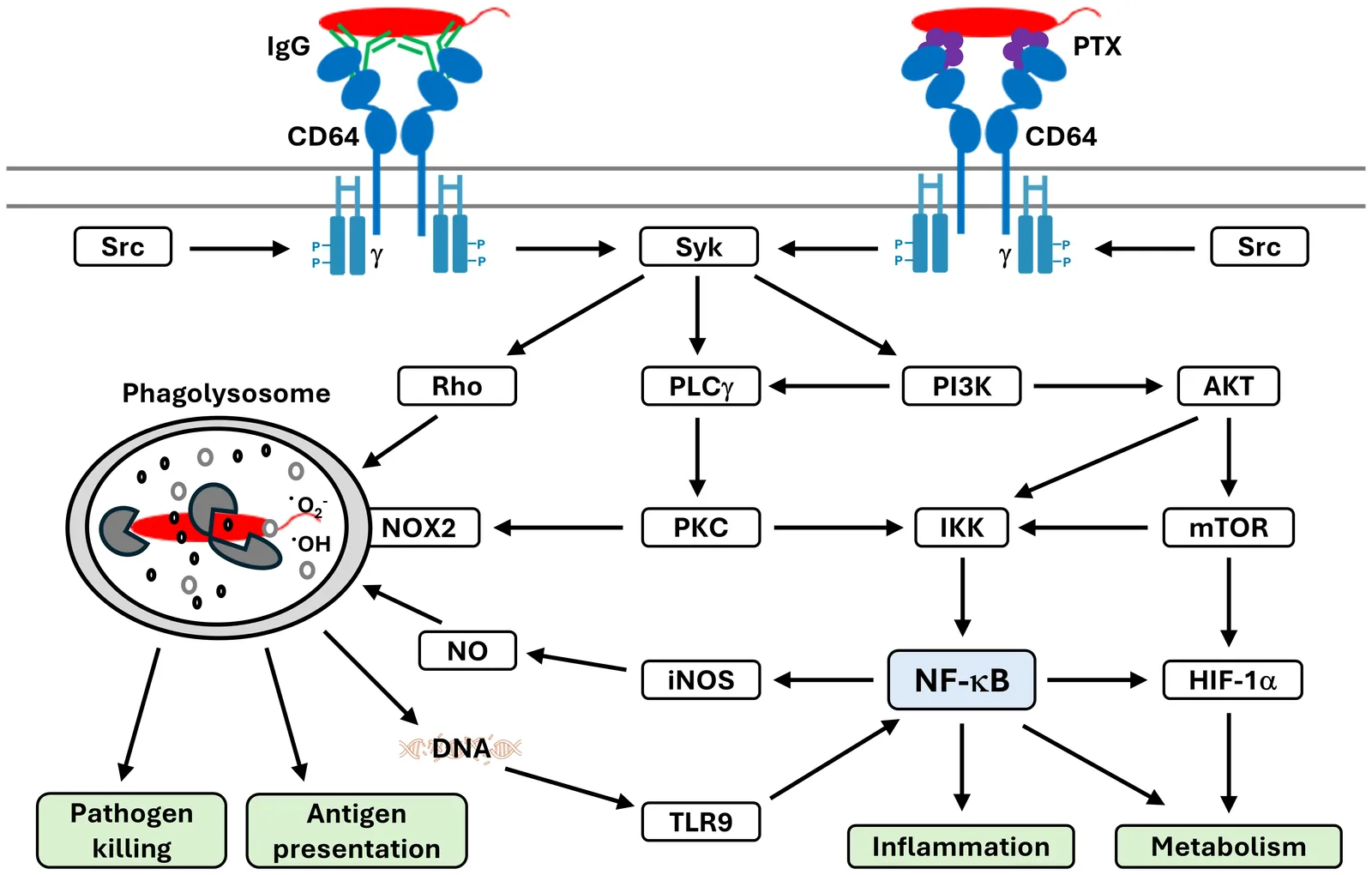
Biological roles and molecular mechanisms of CD64 activation by IgG and pentraxin.

- Phagocytosis, lysosomal fusion, and pathogen killing: The core Syk/PI3K platform coordinates downstream Rho-family GTPases (Rac1 and Cdc42) to drive rapid actin polymerization for physical phagocytic cup formation, routing the captured pathogen into a nascent phagosome and subsequent fusion with lysosomes, exposing the invader to intensive vacuolar acidification and proteolytic degradation for destruction (63–66). Simultaneously, the superior Syk/PI3K node rapidly assembles the multi-subunit nicotinamide adenine dinucleotide phosphate (NADPH) oxidase 2 (NOX2) complex directly at the phagosomal membrane (63–66). This immediate, high-intensity respiratory burst floods the phagosome with toxic superoxide anions and hydrogen peroxide to ensure absolute pathogen destruction, while parallel IKK/NF-κB signaling activated by the Syk/PI3K node generates a subsequent transcriptional spike in inducible nitric oxide (NO) synthase (iNOS) for prolonged reactive nitrogen species (RNS) generation (67).
- Antigen presentation and DNA exposure: As a direct byproduct of this vacuolar destruction, the digested microbe is broken open, exposing hidden pathogen DNA to engage endosomal TLRs (such as TLR9), which in turn activates the IKK/NF-κB pathway (57, 58, 68). And the liberated peptide fragments are successfully processed and routed directly to major histocompatibility complex (MHC) molecules, initiating antigen presentation to instruct downstream adaptive T- and B-cell networks (66).
- Inflammation: Upon opsonin clustering, the common γ-chain core cross-activates and synergizes with parallel cell surface and endosomal PRRs (69). Their common SYK/PI3K axis plugs to activate several signaling pathways, and especially, the master inflammatory transcription factor NF-κB to mediate the expression of standard pro-inflammatory genes such as TNF-α and IL-1β (70–72). The high-density phosphorylation of the common γ-chain ITAMs induced by CD64 engagement also provides an optimal docking platform for phospholipase C-γ (PLCγ) (73). Once phosphorylated by Syk, PLCγ hydrolyzes membrane PIP_2_ into inositol 1,4,5-trisphosphate (IP_3_) and diacylglycerol (DAG). IP_3_ binds to its receptors on the endoplasmic reticulum, triggering a massive, rapid release of intracellular calcium (Ca^2+^) stores into the cytosol. This initial wave opens plasma membrane CRAC (Calcium Release-Activated Calcium) channels, inducing a sustained, high-amplitude extracellular calcium influx. This profound influx further activates protein kinase C (PKC) and NF-κB and related signaling pathways to fuel biological effects, including abrupt spike in pro-inflammatory cytokine secretion (73, 74).
- Metabolic Reprogramming: PI3K also drives the AKT/mTOR/HIF-1α signaling pathway, in addition to activating NF-κB to induce the expression of hypoxia inducible factor 1α (HIF-1α) and several other metabolism-related genes such as glucose transporter 1 (GLUT1) and pyruvate kinase muscle isozyme M2 (PKM2) (75, 76). This coordination causes cell metabolism to be dominated by aerobic glycolysis, providing the rapid energy fuel required to sustain phagocytosis, clear opsonized pathogens, and support immediate downstream effector functions (20).
- The γ-chain-independent CD64 trafficking motif (silent antigen sampling): Distinct from the canonical ITAM pathway, the short cytoplasmic tail of CD64 contains an autonomous, tyrosine-independent sequence rich in serine/threonine residues (45–47, 77, 78). This sequence permits CD64 to initiate internalization and direct cargo trafficking completely independently of common γ-chain signaling or proximal tyrosine kinase activity. Serving as a shared pathway for both low-density IgG and pentraxin matrices, this motif provides a non-phlogistic route to ferry bound antigens directly into specialized late endosomes and major histocompatibility complex (MHC) class II-containing compartments, facilitating routine homeostatic adaptive surveillance without triggering explosive, toxic cell activation (77, 78).

### 3.2 Unique non-classical signaling as a PRR

CD64 could operate independently of antibodies, pentraxins or other opsonins as a standalone, direct PRR tissue sentinel:

- The γ-chain-independent, direct PRR disconnect: It was observed that CD64 could directly bind to the OmpA of large bacterial invaders, driving macrophage phagocytosis to internalize the whole bacterium (41). Despite each *E. coli* cell expresses around 100,000 molecules on its outer membrane (51), however, this high-density engagement was also reported to fail to induce the cross-linking of CD64 or the recruitment of the γ-chain (41). Instead, the interaction yielded atypical pattern of tyrosine phosphorylation of macrophage proteins, alongside with the suppression of complement receptor (CR3) and TLR4, and the enhancement of CD64, TLR2, inflammatory cytokine induction, and NO production (41). In this described signaling model, while the macrophage successfully engulfed the unopsonized invader, it could not drive lysosomal maturation and killing, transforming the vacuole into a protected, replication-permissive intracellular niche (40).

## 4 CD64 as the driver of trained immunity establishment

The signaling networks detailed above do more than immediate pathogen clearance; they trigger extensive chromatin remodeling—marked by elevated H3K4me3 and H3K27ac—and shift the cellular energetic baseline toward aerobic glycolysis (20, 38, 58). This functional reprogramming induces a state of transcriptional vigilance, enabling macrophages to mount robust, non-specific secondary protection against heterologous infections (20). As a receptor restricted to and highly expressed on myeloid lineages, CD64 is further upregulated during the early, antibody-naive phase of infection, positioning macrophages as superior anti-infective sentinels (10–19). These attributes thus may arm CD64 as a primary coordinator of this innate immune memory phenotype.

Yet, the role of CD64 and its circulating pentraxin ligands in orchestrating innate immune memory has been completely overlooked. Existing literature treats the upregulation of both CD64 and CRP merely as passive hallmarks of the trained phenotype (79). This passive view contrasts sharply with classical PRRs, such as Dectin-1, TLR2, and TLR4, whose mechanistic requirements in macrophage memory have been extensively studied, albeit primarily using purified components like β-glucan and LPS as priming agents (80–83). Notably, even within these single-component models, genetic ablation studies challenge the absolute necessity of these canonical PRR pathways. Knockouts of Dectin-1, TLR2, or both demonstrate that β-glucan can induce macrophage innate memory independently of its classic receptor complex (82). Similarly, TLR4-knockout studies reveal a major, but not absolute, requirement for TLR4 in LPS-induced memory (83). These ostensibly surprising findings challenge the conventional paradigm and highlight the importance of other PRRs and/or partners. Because the induction of trained immunity is entirely antibody-independent, circulating PRRs, such as CRP and SAP, are particularly critical in bridging early microbial recognition to the cell surface. By acting as the physical deployment platform for these innate opsonins, CD64 may serve as a central engine driving trained immunity (**Figure 2**).

**Figure 2:**
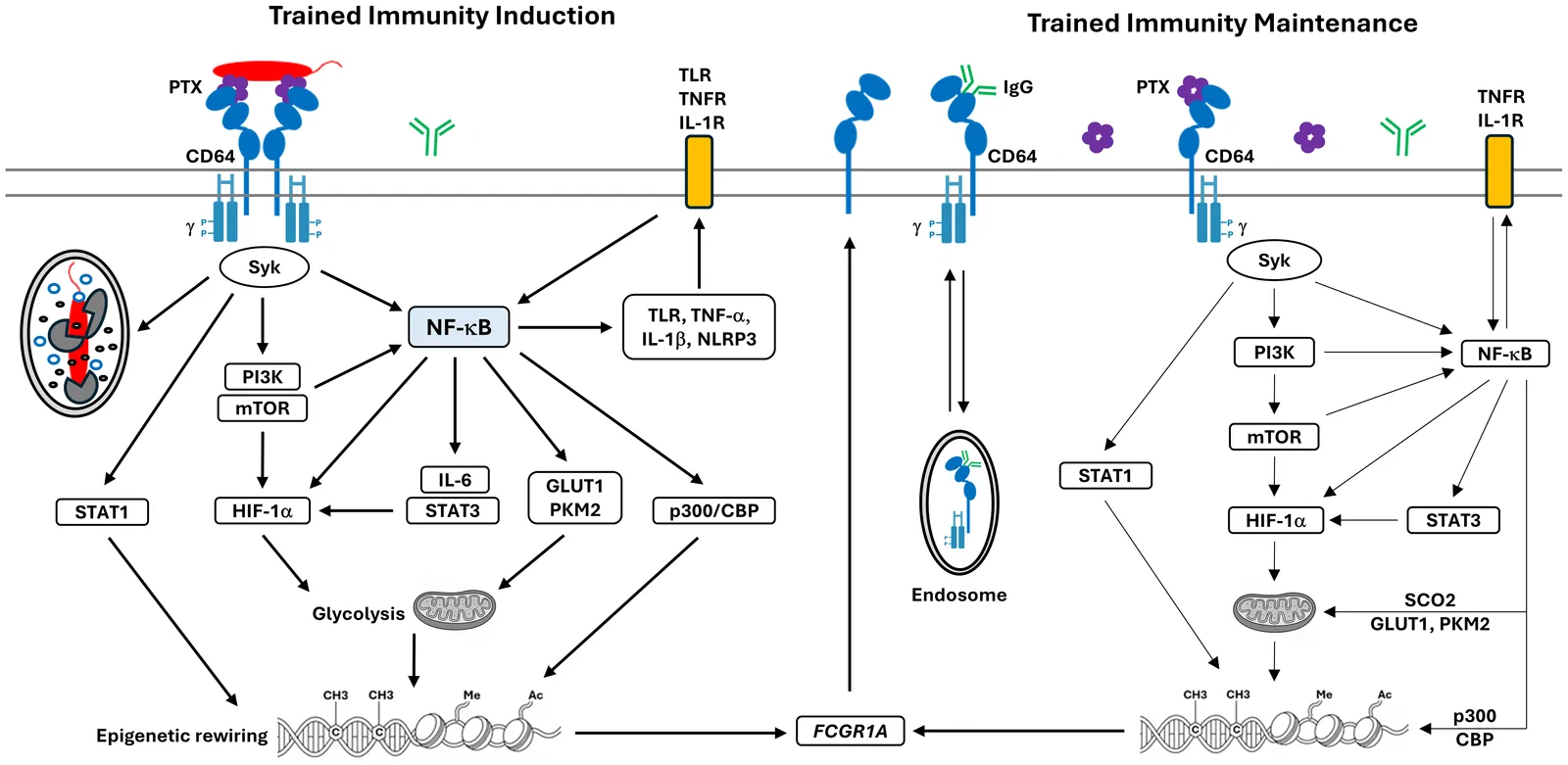
Mechanistic roles of CD64 in trained immunity.

### 4.1 The induction phase (Priming the primary signal and fuel)

Innate immune memory induction requires a primary stimulus to flip the metabolic switch of a resting myeloid cell, a checkpoint mediated by baseline CD64 expression on naive macrophages. Upon encountering an initial antibody-independent pathogen insult, humoral pentraxins like CRP cross-link with CD64 (22, 31). This cross-linking initiates lateral trans-activation with adjacent membrane PRRs and receptors (58, 69). This co-ligation triggers a robust signaling wave through shared proximal Syk and PI3K signaling modules, driving the normoxic stabilization of HIF-1α via the mTORC1 pathway (38, 58, 69). This precise pathway serves as the core metabolic catalyst to establish trained immunity, instantly shifting the cell from oxidative phosphorylation to high-rate aerobic glycolysis—the Warburg-like metabolism that is required to sustain heightened cellular alertness (20).

### 4.2 The imprinting phase (Providing the epigenetic memory)

The metabolic flux initiated by non-classical CD64 signaling directly drives the epigenetic rewiring needed to secure the trained phenotype. This rapid glycolytic switch disrupts the mitochondrial tricarboxylic acid (TCA) cycle at critical nodes, forcing a massive accumulation of cellular metabolites, specifically succinate and fumarate (20, 84–91). These accumulated intermediates translocate to the nucleus, where they act as competitive inhibitors of Jumonji-domain-containing histone demethylases (KDMs). By blocking KDM-mediated erasure—particularly via fumarate-mediated inhibition of H3K4 demethylases—the cell preserves and deposits permissive histone marks, specifically H3K4me3 and H3K27ac, directly at host-defense gene promoters, establishing the physical epigenetic “scars” of innate memory (20).

### 4.3 Orchestrating role of NF-κB autoactivation loop in trained immunity establishment

- Direct NF-κB reinforcement: Syk/PI3K/mTOR/HIF-1α signaling reprograms metabolism and epigenetics, but NF-κB acts as the master orchestrator (20, 75, 92–95). Activated by pentraxin/CD64/Syk/PI3K cascades alongside TLRs, NF-κB upregulates the expression of HIF-1α, GLUT1/SLC2A1, and PKM2, thereby reinforcing glycolytic flux. Concomitantly, NF-κB recruits histone acetyltransferases (HATs), such as p300/CBP, to its target promoters, driving the local deposition of H3K27ac marks and transforming latent enhancers into active, hyper-accessible hubs (96–98). Interestingly, NF-κB maintains its own persistent firing, especially via an autocrine IL-1β/TNF-α cytokine loop (68, 72)
- Synergistic STAT1 and STAT3 axis: NF-κB also induces the secretion of IL-6, which subsequently activates STAT3 signaling (96–106). This pathway acts as a critical homeostatic rheostat, optimizing mitochondrial respiration and electron transport chain efficiency for long-term survival energy while balancing sustained NO production. Simultaneously, the Syk-phosphorylated STAT1 transcription factor complex operates in parallel to secure epigenetic continuity; it translocates to the nucleus to enforce sustained H3K4me3 marks at targeted host-defense gene loci, preventing chromatin compaction and maintaining open, long-term transcriptional permission (97, 107, 108). Together, this STAT1/STAT3 axis ensures that the macrophage possesses both the metabolic endurance and the architectural epigenetic blueprint required to execute memory responses.
- Nod-like receptor (NLR) coordination: Through the coordinated actions of NF-κB and the STAT axes, the non-classical CD64 core establishes the essential transcriptional priming of the cell, driving the expression of structurally inactive *IL1B*, *IL18*, and the PRR *NLRP3* (109–111). In parallel, it orchestrates pathogen engulfment and internalization. This phagocytic routing delivers pathogens directly into the phagolysosomal pathway, facilitating the intracellular release of unmasked PAMPs to engage and activate cytosolic NLRs, including NOD2 (nucleotide-binding oligomerization domain 2) and NLRP3 (109–111). Once activated, NOD2 amplifies secondary waves of NF-κB transcription, while NLRP3-mediated caspase-1 activation processes and unleashes mature IL-1β and IL-18. Secreted IL-1β binds to surface IL-1R in a potent autocrine loop, fueling signals that permanently lock the downstream NF-κB and mTORC1/HIF-1α glycolytic engine into place.

## 5 CD64 as the maintainer and executor of trained immunity

Following the establishment of trained immunity and pathogen clearance, downstream signaling pathways—including NF-κB and STAT3—attenuate from their peak to a modest baseline higher than pre-challenge levels via two distinct mechanisms. First, the absence of antigen forces CD64 to switch from highly stimulatory pathogen-pentraxin complexes to free pentraxins and monomeric IgGs. Because these free ligands are only weak activators, receptor firing drops significantly (112–116). Second, this reduction is reinforced by internal negative feedback loops that tightly regulate and dampen the acute signaling cascade (117–123). Together, these mechanisms downregulate acute inflammation but also prevent the macrophage from returning to its original naive state, particularly given the significant upregulation of CD64 in trained cells compared to naive ones (12–19, 97, 98, 124). Instead, the macrophage settles at a new, elevated homeostatic baseline, utilizing persistent metabolic and epigenetic wiring to maintain constant transcriptional alertness for a secondary encounter.

### 5.1 Monomeric IgG-mediated CD64 dynamics: low-tone signaling and active receptor redeployment

Because monomeric IgG binds to CD64 without cross-linking the receptor, it fails to induce the multivalent clustering required to fully activate downstream signaling cascades (112–116). However, the ITAMs on the associated FcR γ-chain are transiently exposed, allowing the unclustered γ-chain to deliver a low-amplitude tonic signal that maintains homeostasis. More importantly, the gentle signal induces a unique structural shift: CD64 rapidly dissociates from the actin-binding protein filamin (112–116). This structural uncoupling frees the complex for endocytosis. Because the cargo lacks multivalent cross-linking, the complex bypasses lysosomal degradation pathways (114–116). Instead, the internalized CD64-lgG complex enters a highly efficient recycling loop, returning to the cell surface in a fresh, fully competent state for immediate surveillance (114–116).

### 5.2 Free pentraxin-induced baseline CD64 activation: tonic signal and sub-threshold co-ligation

In parallel, circulating free pentraxins CRP and SAP serve as a complementary humoral arm to sustain the trained phenotype without triggering a full inflammatory response. Mirroring monomeric IgG, free pentraxins cannot cluster multiple receptors due to a strict 1:1 binding stoichiometry (31). In the absence of a pathogen, free pentraxins induce a steady baseline signal to maintain the new homeostasis while simultaneously creating a weak, sub-threshold co-ligation with neighboring membrane PRRs to prime cells against reinfections (40).

### 5.3 Controlled autocrine loops and feedforward axes: locking in the high basal equilibrium

Following pathogen clearance, feedback inhibition weakens but does not fully silence the NF-κB and STAT3 signaling pathways. This attenuation coordinates with permanently upregulated CD64 and the autocrine loops detailed in Section 4.3 to establish a new homeostatic steady state. This cooperative axis preserves a persistently elevated basal pool of active NF-κB and STAT3 while shifting the transcription factor profile from predominantly pro-inflammatory to mostly inflammation-resolving, ultimately stabilizing into a delicate new balance. This newly balanced baseline activity maintains a hybrid metabolic state where continuous oxidative phosphorylation (OXPHOS) and glycolysis coexist, ensuring a steady production of the intracellular metabolites required to block histone demethylases. In this regard, NF-κB also induces macrophage expression of synthesis of cytochrome c oxidase 2 (SCO2), a mitochondrial enzyme critical for OXPHOS (125). By physically preventing the erasure of permissive epigenetic marks, this ongoing molecular conversation secures a state of absolute readiness for rapid execution upon a secondary challenge.

## 6 CD64 as the self-sustaining engine regulated at both RNA and protein levels

Most strikingly, the chromatin uncoiling in primed cells is the *FCGR1A* (CD64) gene locus itself. Because its promoter is left wide open during the imprinting phase, the cell is locked into a state of persistent CD64 hyper-expression. While maintaining a state of innate immune memory, this creates a hyper-reactive recall loop: when the trained macrophage encounters a secondary pathogen or immune complex, the over-expressed surface CD64 dictates a drastically lowered activation threshold. The cell rapidly deploys its pre-primed core engines, yielding the accelerated phagocytosis and explosive cytokine output (*IL-6, TNF-α, IL-1β*) mediated by NF-κB and STATs that characterize the trained state.

### 6.1 Promoter remodeling and RNA transcription

CD64 expression is largely restricted to myeloid cells (10, 11). Under homeostatic conditions, resting macrophages maintain a poised, basal chromatin state at the *FCGR1A* promoter, which lacks a canonical TATA box but contains an essential PU.1-binding motif along with conserved gamma-activated sequences (GAS) and interferon-stimulated response elements (ISRE) (10, 126–128). This TATA-less promoter is regulated by baseline histone deacetylase (HDAC) activity, maintaining basal acetylation levels that permit rapid activation. Upon stimulation with IFN-γ, phosphorylated STAT1 dimers bind the GAS element and recruit HATs, specifically CBP/p300, driving the deposition of permissive H3K27ac marks and robust transcriptional activation (10, 126–128).

### 6.2 Protein stabilization

Interestingly, CD64 requires both the common γ chain and filamin A (FLNA) to be optimally expressed on the cell surface (129, 130). While the exact mechanism underlying CD64 surface expression remains fully to be elucidated, it is highly plausible that FLNA binds to the short cytoplasmic tail of CD64. Together with the common γ chain, this interaction anchors CD64 to the cortical actin cytoskeleton at the cell surface, shielding it from default routing to lysosomes for degradation. Highly consistent with the potential roles of CD64 in trained immunity, both the γ chain and FLNA are correlatedly increased following macrophage activation (129, 130).

## 7 Conclusion and future perspective

CD64 may function as a central hub orchestrating immunity and innate immune memory. When pathogens invade—and before adaptive immunity is initiated—it acts as a PRR, either directly or via pentraxins, to capture pathogens and execute phagocytosis. Operating in tight tandem with other PRRs like TLRs, it initiates inflammation and kills the pathogen while simultaneously presenting processed antigens to prime adaptive immunity. Concurrently, it routes signaling networks to reprogram metabolic and epigenetic platforms for a new homeostatic baseline within the cells, including a higher expression of CD64 and enhanced phagocytic ability. By dynamically binding to pentraxins or monomeric IgG after pathogen killing and trained immunity induction, CD64 persistently maintains these metabolic and epigenetic alterations, sustaining a state of primed epigenetic poise upon a subsequent homologous or heterologous challenge.

While these mechanisms warrant further investigation and validation, unraveling these complex, multi-tiered networks will be crucial for developing targeted therapeutics capable of overcoming immune tolerance or breaking self-sustaining inflammatory circuits in chronic autoimmune, infectious, and inflammatory diseases. Given the long-term nature of the innate immune memory that was initially recognized in the 1940s across species from plants to invertebrate animals to mammals and humans, using modern technologies to link this phenomenon to epigenetic and metabolic programming in myeloid cells is logical yet fascinating (20, 21, 96–98, 131–143). However, unlike CD64, epigenetic and metabolic rewiring do not act as the primary drivers or direct executors of trained immunity. In contrast to permanent genetic alterations, epigenetic and metabolic changes are global and reversible, a fundamental characteristic that is physiologically vital across all eukaryotic cellular systems (88, 89, 144). Attempting therapeutic translation by rewiring these broad, universal mechanisms—or targeting ubiquitous hubs involved in trained immunity such as NF-κB, STAT3, and cytokines like IL-1—carries a high risk of inducing systemic toxicity or inadvertent immune tolerance, leaving patients highly vulnerable to tissue injury and secondary infections, even if nanoparticle technologies designed to target macrophages can partially mitigate these challenges (145–151). This risk is underscored by the fact that historical exposures (infections and vaccines) can train macrophages toward life-saving non-specific protection, or cause them to lapse into maladaptive hyper-inflammation or tolerance (151).

Shifting the therapeutic focus away from broad metabolic and epigenetic programming toward precise, myeloid-specific mechanisms like CD64 may provide a concrete therapeutic target and a predictive biomarker to enhance immunity against life-threatening infections. However, given CD64’s pleiotropic roles, its therapeutic potential—such as through CD64-chimeric macrophages—must be carefully balanced against the risk of toxicity. Indeed, aberrant over-expression of CD64, pentraxins, and IgG has already been implicated in the pathogenesis of certain autoimmune diseases, such as systemic lupus erythematosus (SLE) and rheumatoid arthritis (RA) (152–158).
